# Ligand cluster-based protein network and ePlatton, a multi-target ligand finder

**DOI:** 10.1186/s13321-016-0135-5

**Published:** 2016-04-30

**Authors:** Yu Du, Tieliu Shi

**Affiliations:** Center for Bioinformatics and Computational Biology, Shanghai Key Laboratory of Regulatory Biology, Institute of Biomedical Sciences and School of Life Sciences, East China Normal University, Shanghai, 200241 China

**Keywords:** Protein network, Polypharmacology, Promiscuity, Ligand, Pathway, Gene ontology

## Abstract

**Background:**

Small molecules are information carriers that make cells aware of external changes and couple internal metabolic and signalling pathway systems with each other. In some specific physiological status, natural or artificial molecules are used to interact with selective biological targets to activate or inhibit their functions to achieve expected biological and physiological output. Millions of years of evolution have optimized biological processes and pathways and now the endocrine and immune system cannot work properly without some key small molecules. In the past thousands of years, the human race has managed to find many medicines against diseases by trail-and-error experience. In the recent decades, with the deepening understanding of life and the progress of molecular biology, researchers spare no effort to design molecules targeting one or two key enzymes and receptors related to corresponding diseases. But recent studies in pharmacogenomics have shown that polypharmacology may be necessary for the effects of drugs, which challenge the paradigm, ‘one drug, one target, one disease’. Nowadays, cheminformatics and structural biology can help us reasonably take advantage of the polypharmacology to design next-generation promiscuous drugs and drug combination therapies.

**Results:**

234,591 protein–ligand interactions were extracted from ChEMBL. By the 2D structure similarity, 13,769 ligand emerged from 156,151 distinct ligands which were recognized by 1477 proteins. Ligand cluster- and sequence-based protein networks (LCBN, SBN) were constructed, compared and analysed. For assisting compound designing, exploring polypharmacology and finding possible drug combination, we integrated the pathway, disease, drug adverse reaction and the relationship of targets and ligand clusters into the web platform, ePlatton, which is available at http://www.megabionet.org/eplatton.

**Conclusions:**

Although there were some disagreements between the LCBN and SBN, communities in both networks were largely the same with normalized mutual information at 0.9. The study of target and ligand cluster promiscuity underlying the LCBN showed that light ligand clusters were more promiscuous than the heavy one and that highly connected nodes tended to be protein kinases and involved in phosphorylation. ePlatton considerably reduced the redundancy of the ligand set of targets and made it easy to deduce the possible relationship between compounds and targets, pathways and side effects. ePlatton behaved reliably in validation experiments and also fast in virtual screening and information retrieval.

**Graphical abstract:**
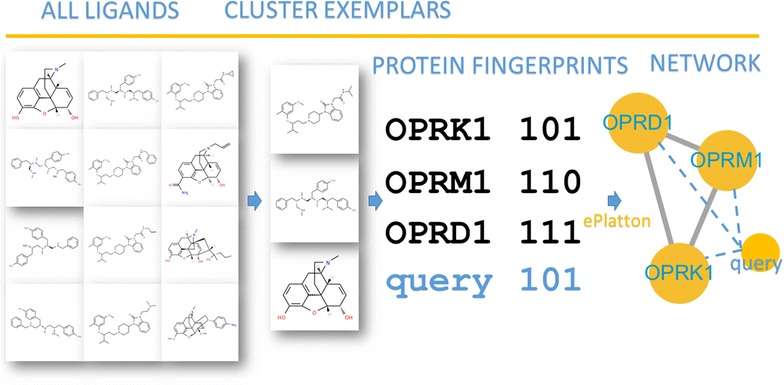
Cluster exemplars and ePlatton’s mechanism.

**Electronic supplementary material:**

The online version of this article (doi:10.1186/s13321-016-0135-5) contains supplementary material, which is available to authorized users.

## Background

The physicochemical properties, sequence information and crystal structures have been used to study the protein function and the relationship between proteins [1–3]. With the protein-based information accumulating, millions of ligand–protein interaction records have been deposited in the open ligand databases for the past decade. These interaction data enable us to explore the properties of proteins from the perspective of ligand. The hypothesis that similar molecules should exhibit similar biological activities is generally valid and wildly used in the field of drug design [4]. At the same time, the protein network can illustrate biological information in a more powerful and effective view, which could not be easily achieved by multiple alignments and large trees [5]. So ligand-based protein network could be a stimulating way for finding drug-like compounds and protein classification.

As far as we know, there are generally two kinds of methods to build the ligand-based protein network. The first is represented by drug-target binary association [6], this helps us find not only that one target may interact with multiple drugs and one drug may interact with multiple targets but also that proteins from different gene families may link each other at the ligand level. The second is by means of similarity between ligand sets that functionally regulate their targets [7]. According to the ligand-related e-value of different proteins, proteins can be grouped and related at the ligand level. A conceptionally similar but technically different approach is based on Shannon Entropy Descriptors (SHED) derived from distributions of atom-centred feature pairs extracted from the topology of molecules [8]. However, the ligand set recognized by a certain target can be composed of different molecular types. For example, Fentanyl, Naltrexone, Alvimopan and Dezocine, four FDA-approved drugs [9], all target Mu opioid receptor (OPRM1), but they share few similarities in the 2D structure, although both Naltrexone and Alvimopan are antagonists [data source: ChEMBL_19]. Perphenazine, Pimozide and Risperidone, three antagonists of D2-like dopamine receptor (DRD2), also differentiate each other in 2D structure. We believe that it should be more rational to distinguish proteins by the 2D-structure clusters of ligands.

Here, we introduce a method to quantitatively distinguish and relate proteins by ligand clusters. For numerous ligands, we can cluster them by the similarities of their 2D structures. We presume that if two proteins share more ligand clusters, they are more similar at the ligand and ligand cluster level. Therefore, proteins can be annotated by the ligand clusters they recognize. The similarity of two annotated proteins can be represented by Jaccard index [10]. Then, we display the ligand cluster- and sequence-based protein similarity in LCBN and SBN and compare and cluster both networks. Our results underscore the biological and chemical meanings of protein modules in the LCBN and the ePlatton, a web platform with integrated information, provides a way to take advantage of the ‘good’ polypharmacology and get rid of the ‘bad’ one.

## Results

### Quantifying the ligand cluster- and sequence-based protein similarity

We extracted 234,591 relatively strong interactions between 156,151 distinct ligands and 1477 proteins from ChEMBL [11], an informative public database containing a wealth of drug-like bioactive compounds. Of these proteins, 231 gene families were covered, including 194 G protein-coupled receptors, 44 solute carriers and 34 nuclear hormone receptors. On average each protein target had 159 ligands, with a median of 24.

Firstly, each ligand was converted from its 2D structure to the CDK [12] (Chemistry Development Kit) 2048-bit fingerprint. The similarity between each fingerprint could indicate the similarity of the 2D structure of each ligand. Then, we use affinity propagation method [13] to cluster the ligands by the similarities of each pair of fingerprints. As a result, we gained 13,769 clusters, each of which included from 113 to 2 2D-structure similar ligands or a single ligand that could not find a cluster mate. Finally, we distinguished proteins by the mutual recognition pattern of proteins and ligand clusters. We presume that if two proteins share more ligand clusters, they are more similar at the ligand cluster and ligand level. By this means, we quantified the relationship between each pair of proteins by ligand-cluster level Jaccard similarity coefficient [10], i.e. at the level of ligand. The protein sequence similarity was converted from the global sequence distance obtained by ClustalO [14].

**Fig. 1:**
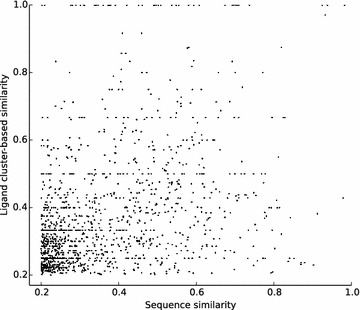
The comparison of ligand cluster-based similarity and global sequence similarity

Next, we compared the two kinds of similarities and found that there was no correlation between them even at the high similarity level and that points whose both similarities were below 0.2 consisted of 99 % of the all (Fig. 1).

### Construction and topological analysis of the chemical and biological protein networks

**Fig. 2:**
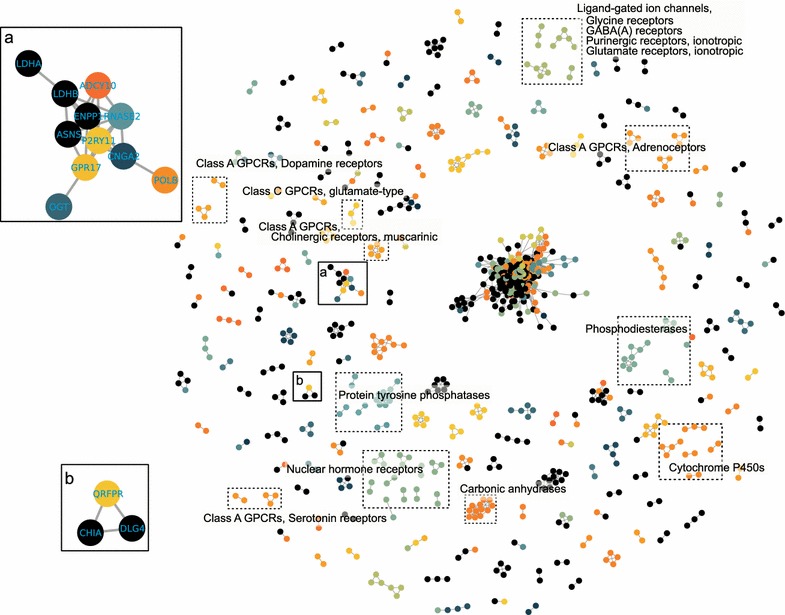
The ligand cluster-based network. *Edges* represent the ligand cluster-based similarity. This network only display the similarities above 0.25. Detailed *box* **a** and **b** are shown with gene names. *Different colours* represent different HGNC gene families

The ligand cluster-based network illustrated both intra-connections between proteins in the same gene family and inter-connections between members from different gene families (Fig. 2). This meant that identical or similar ligands were not shared only in an individual gene family but also between different gene families. Of a total of 828 proteins in this network, 549 had over one link to other proteins and 240 proteins formed a giant component, suggesting that the commonality between proteins at the level of ligand should result from the chemical crosstalk between them. The number of ligand clusters that interacted with an individual protein varied differently. Although two ion channel proteins (i.e. KCNH2 and CACNA1H) and MCL1 ranked at the top three in the 1477-protein list, sorted by the number of ligand clusters in descending order, none of them were included in the network because of the displaying criterion that protein similarities should be over 0.25. Furthermore, there was no correlation between the number of interacting ligand clusters and the degree of each protein (Spearman correlation coefficient was 0.06) [see Additional file 1], indicating that the number of interacting ligand clusters was not a determinant for ligand-based similarities between proteins. For example, however, DRD2, CNR2 and CNR1 interacted with 409, 403 and 392 ligand clusters, their degrees in the network were respectively 2, 1 and 1. The degree distribution of the network showed that most proteins only linked a few proteins [see Additional file 2], whereas a small quantity of proteins such as three proteins from the family of mitogen-activated protein kinase cascade (i.e. MAP4K3, MAP4K1 and MAP4K5), one serine/threonine kinase (i.e. STK25) linked to more than eighty proteins.

**Fig. 3:**
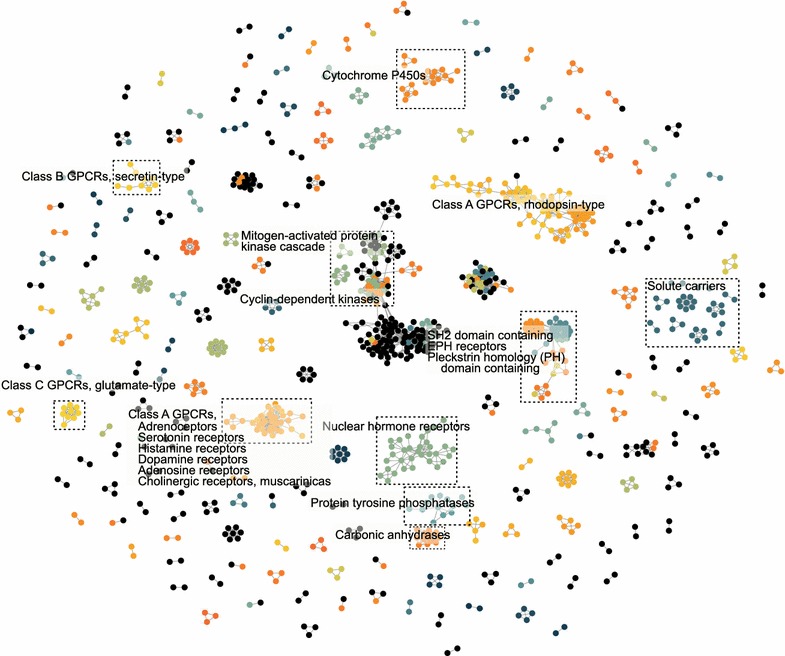
The sequence-based network. *Edges* represent the global sequence similarity. This network only display the similarities above 0.25. *Colour* coding is the same as Fig. 2

The meaning of sequence-based network was more explicit (Fig. 3). Two proteins were linked when their global sequence similarity was above a threshold. Of a total of 1025 proteins in the network, 786 had more than one link to other proteins and 165 proteins formed a giant component. Although the numbers of connected components in both networks were similar, that was 191 for ligand cluster-based network and 199 for sequence-based network, the detailed size and members of each component were not that similar. Next, we would compare and cluster both networks.

### Comparing the ligand cluster- and sequence-based networks

In the sequence-based network, most proteins linked to and clustered with the same gene family members with expectations. Obviously, G protein-coupled receptor family, whose seven-transmembrane domain made itself different from most other gene families, formed 18 nearly exclusive subnetworks, including 15 Class A subnets, 2 Class B subnets and 1 Class C subnet. All members in GPCR giant subnet belonged to GPCR/Class A although HGNC did not annotate UTS2R as GPCR, and in the giant subnet, all chemokine receptors, neuropeptide receptors and opioid receptors clustered together (Fig. 3). Meanwhile, all cyclin-dependent kinases, dozens of mitogen-activated protein kinases and several pleckstrin homology (PH) domain containing proteins consisted of almost thirty percent of the giant component of the whole network and the other seventy percent belonging to HGNC undefined family were protein kinases, suggesting that proteins involved in the signal transduction bore some structural or domain resemblance (Fig. 3).

Compared with the sequence-based network, large components in the sequence-based network were split into smaller ones in the ligand cluster-based network. For example, the neuropeptide receptors, opioid receptors and somatostatin receptors, most members of which clustered in a single community in the SBN, separated from each other and formed several tiny clusters in LCBN. If we zoomed in for a close-up of some subnets, we could find some unusual associations. For instance, sub-cluster a (Fig. 2a) consisted of LDHB, P2RY11, RNASE2, ENPP1, ADCY10, GPR17, CNGA2 and ASNS which were highly intra-connected although these eight proteins belonged to distinct families and the maximum of global sequence similarities between them was 0.22 (i.e. P2RY11 and GPR17). CHIA, DLG4 and QRFPR formed a cluster in LCBN sub-cluster b (Fig. 2b) although the maximum of their global sequence similarities was 0.11 and none of them met the display criterion for SBN.

Although communities in both networks were formed independently from any knowledge of known gene families, the generated major subnetworks visibly clustered as gene families. By affinity propagation method, 263 and 305 clusters emerged from LCBN and SBN. Then we used normalized mutual information to compare the clusters among both networks and the HGNC classification. Interestingly, two parallel construction process resulted in being around 72 % similar to the HGNC classification and around 91 % similar to each other [for detailed cluster information and comparison of cluster methods, see Additional file 3]. Next, from the perspective of ligand cluster, we dissected the target promiscuity, ligand cluster promiscuity and explained the high intra-connection of the LCBN giant component.

### The promiscuity of ligand clusters and targets underlying the LCBN

**Fig. 4:**
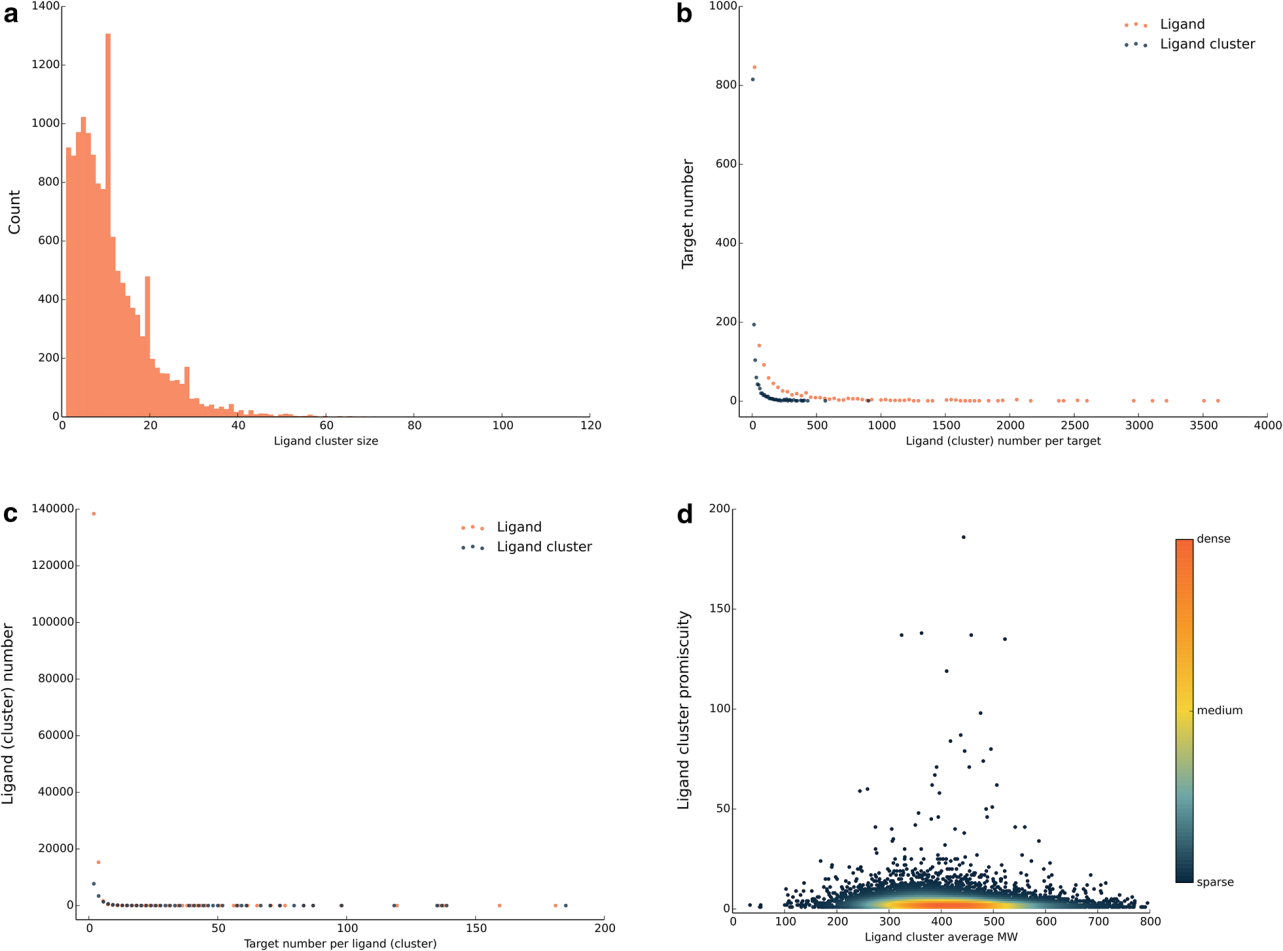
The promiscuity of ligand clusters and targets underlying the LCBN. **a** The histogram of ligand cluster size, **b** the target distribution of ligands and ligand clusters, **c** the ligand (cluster) distribution of targets, **d** the relationship between the average molecular weight of ligand clusters and the ligand cluster promiscuity, nodes are *coloured* by the number of the nearby nodes, i.e. node density

**Fig. 5:**
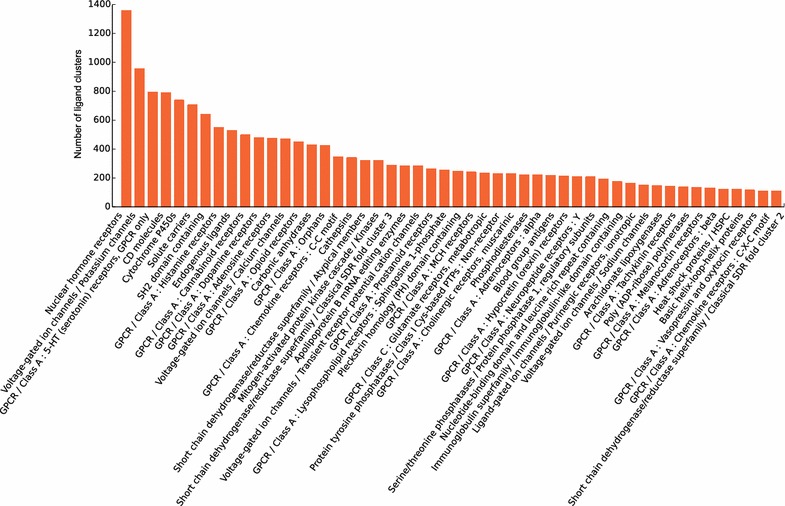
The ligand cluster number of HGNC gene family. The *bar graph* of non-redundant ligand clusters of members in each HGNC gene family, fifty families in descending order of the ligand cluster size

First, the histogram of the ligand cluster size showed that after the ligand cluster size of 5, the number of ligand clusters at certain ligand cluster size decreased as the ligand cluster size increased (Fig. 4a). Interestingly, the obviously high number of ligand clusters appeared around the size of 10, 19 and 28. Next comparing the promiscuity of ligands and ligand clusters, after counting the number of targets that each ligand and ligand cluster interacted with (Fig. 4b), we found that both promiscuity had the same trend and most ligands or ligand clusters had less than 50 targets. In the same way, from the perspective of ligands and ligand clusters, the plot of target promiscuity (Fig. 4c) showed that ligand clusters could considerably reduce the redundancy of ligands and make it easy to find out the ligands with similar structures that interacted with different targets. Last, we showed the relationship between the promiscuity and average molecular weight (AMW) of the ligand clusters (Fig. 4d). The AMW of most ligand clusters was around 400. The degree of promiscuity of light ligand clusters whose AMW ranged from 200 to 500 tended to be greater than the heavy ones and in this molecular mass range, there were several ligand clusters that interacted with more than 50 different targets. We also showed the relationship between the HGNC gene family and the number of ligand clusters, in which the nuclear hormone receptors, potassium channels and 5-HT receptors interacted with the most ligand clusters (Fig. 5).

We next asked why the LCBN’s giant component were highly intra-connected and what biological and chemical property could emerge from the central hairball and the whole LBCN (Fig. 2). In the LCBN’s giant component, there were 240 nodes and 3245 edges, which accounted for 83 % of the total edges of LCBN. Top ten proteins with the highest degrees in the whole network which ranged from 100 to 72 were also in this giant component and from different kinase families (e.g. Mitogen-activated protein kinase cascade and Cyclin-dependent kinase). This consisted with the fact that protein kinases were intensively studied as drug targets and many compounds were designed for the ATP binding sites of these proteins [15]. GO enrichment analysis [16] of the genes showed that many of them significantly associated with phosphorus metabolic process and phosphorylation. In KEGG pathway study [17], 8 out of the top 10 significant pathways were involved in signal transduction, which could be explained by these kinases participating in these pathways [the top 10 GO and KEGG items can be found in Additional file 4]. For the ligands and ligand clusters underlying LCBN, the ligand cluster with the highest protein-interacting number (157) was an 18-ligand cluster represented by Sunitinib [CHEMBL535], an FDA-approved multi-target receptor tyrosine kinase inhibitor. For the full top 10 ligand clusters see Additional file 5.

### ePlatton, a multi-target ligand finder

We integrated the relationship of targets and ligand clusters into our web platform, ePlatton, to help us explore their corresponding promiscuity. We fulfilled three main functions in ePlatton: (1) library design facility, (2) OMIM disease [18], KEGG pathway [17] and SIDER drug adverse reaction [19] conditional search and (3) global protein sequence and ligand cluster similarity search.

First, from our prior ligand cluster range of targets, users can build their own compound libraries guided by the ligand clusters of targets or target sets that they interest. The AP cluster method chose an exemplary ligand from each ligand cluster and the exemplars can be used as prototypes for chemical library design. Second, we related the targets and ligand clusters with the information of diseases, pathways and drug adverse reactions to facilitate the query of ligand clusters associated with certain disease, pathway and drug adverse reaction. By advanced search, users can combine several pathways and/or proteins and exclude other pathways and/or proteins to find the ligands and ligand clusters that binding proteins in the pathways and/or proteins that satisfy the condition. The advanced search strategy also apply to the library design, for example, designing a series of compounds that bind to some proteins but don’t bind to the other proteins. Third, users can submit chemical structure, e.g. SMILES strings, to our platform to search for the similar ligand clusters that their interested compounds belong to. Then, they can deduce the possible relationship between their compounds and the targets, pathways, diseases and side effects.

Besides these main functions, users can exam the target range and 2D structure of every ligand in each ligand cluster. In this way, we considerably reduced the redundancy of the ligand set of targets and established a method to explore the ligand cluster promiscuity. We also included the FANTOM5 gene expression data [20], which could help users to exam their targets across 14 normal tissues and 10 cancer cell lines. For large-scale analyses, the information of targets and ligand clusters can be downloaded from the ePlatton website.

### Retrospective validation and prospective prediction power of ePlatton

**Fig. 6:**
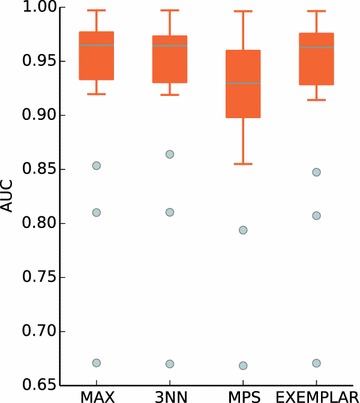
The *box plot* of AUC of four methods for ePlatton validation and comparison

**Fig. 7:**
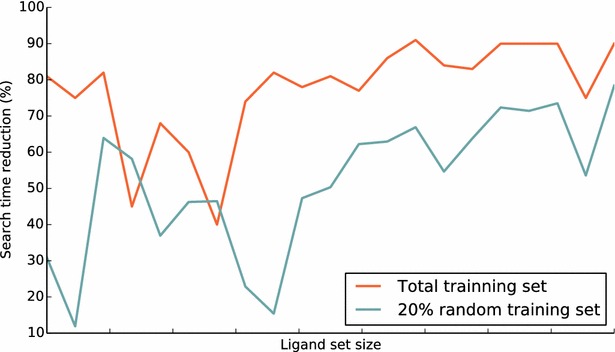
The percentage of search time reduced versus ligand set size from small to big

Several methods of virtual screening based on ligand set have been proposed, such as similarity ensemble approach (SEA) [7], pair-wise similarity (MPS) [21] and other different data fusion [22] rules (e.g. MAX, KNN). We used the directory of useful decoys (DUD) [23] dataset to retrospectively compare ePlatton and data fusion methods. As shown in Table 1 and Fig. 6, MAX, 3NN and EXEMPLAR (i.e. ePlatton) were better than MPS in average AUC and showed no significant differences in post hoc Friedman’s Test [24, 25], which meant ePlatton retained the predictive power of traditional data fusion methods while reducing the time of virtual screen and information retrieval (Fig. 7).

**Table 1:** Average AUC of four methods and the P value of post hoc Friedman’s Test

	MAX	3NN	MPS	EXEMPLAR
Average AUC	0.938913333	0.936745381	0.915506762	0.937318571
P value	MAX	3NN	MPS	EXEMPLAR
MAX				
3NN	6.30E−001			
MPS	7.52E−008	5.94E−005		
EXEMPLAR	1.76E−001	8.37E−001	1.83E−003

**Fig. 8:**
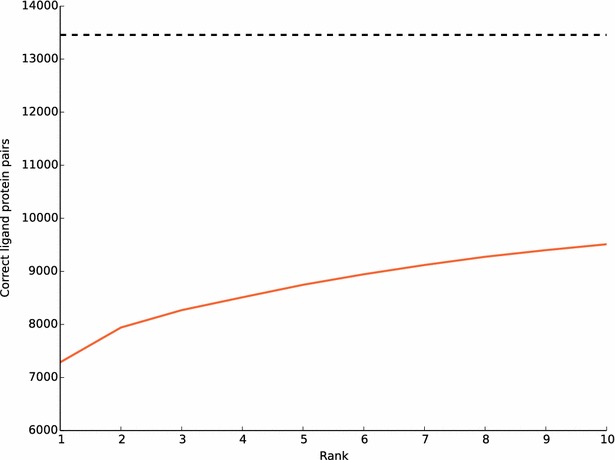
The number of accumulated hit interactions from top 1 to 10. The number of correctly predicted ligand–protein pairs increased from 7287 to 9511. The total number of novel ChEMBL_20 interactions was 13456 as the *dash line* showed

**Fig. 9:**
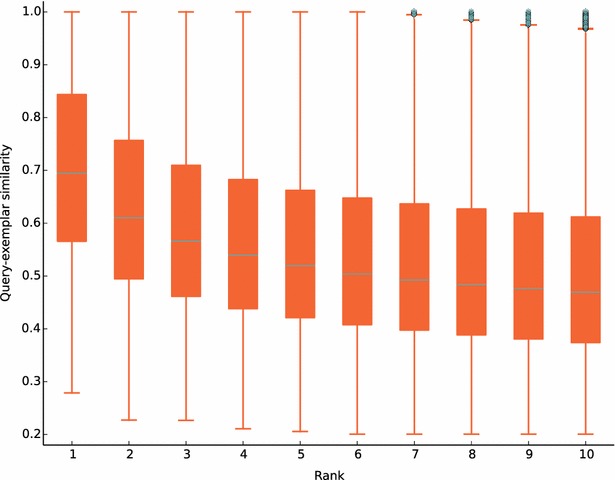
The box plot of query-exemplar similarity of each rank

To evaluate the prediction ability of ePlatton, we extracted 13456 new strong interactions (Ki, Kd, EC50 or IC50 < 10 μM) from the latest ChEMBL (v20) [11], used these compounds as queries to search the ePlatton for similar ligand clusters and checked whether their interacted proteins appeared in the accumulated protein ranges of the 10 most similar ligand clusters. The number of hit interactions increased from top 1 to 10 (Fig. 8). The half (54 %) of total new interactions from ChEMBL (v20) were correctly predicted in the top 1 search result and correct ligand protein pairs from top 10 similarity rank accounted for 71 % of all the new interactions, which showed that the molecular similarity still dominated the way of designing new compound entities. The similarity distribution of each rank was showed in the box plot (Fig. 9) and the median similarity of the top 1 was greater than other ranks. The median decreased from 1 to 10 and the range of similarity became broader.

Here we show two cases in the successful predictions. The first example [26] designed and synthesized a series of 4-phenoxyquinoline derivatives containing pyridazinone moiety; six compounds that further examined showed the c-Met kinase activity in the single-digit μM range. The ePlatton search results included all the compound-MET interactions in the first two most similar ligand clusters and other possible interacted proteins were KIT, KDR and FLT3 [for the detailed list, see Additional file 6]. These proteins play an essential role in cell survival and proliferation [from Function section of their UniProt annotations], which may contribute to the fact that the cytotoxicity of this series of compounds was against A549 cells [26].

In the second example [27], all designed molecules interacted with EGFR, FGFR1 and ABL1 except one selectively targeted tubulin. All the non-selective interactions were predicted by the most similar exemplar of CHEMBL56802 [for the detailed list, see Additional file 7]. This ligand cluster was also annotated by 114 drug adverse reactions, such as acute respiratory distress syndrome, flatulence and myalgia, which could be useful if these molecules were further used for drug development. The ePlatton also integrated the gene expression data, which could be helpful to deduce the degree of different tissues affected by the molecules that users interested.

## Discussion

According to the nature of the object we are looking at there are three possible approaches to mine and dissect the protein–ligand interaction. The study can be focused on targets, bioactive ligands or target-ligand complexes. Recent progress in the ligand-based pharmacology opens new door for virtual screening of high-throughput screening data and predicting polypharmacology and adverse drug reactions [28]. Our method relied on ligand clusters, whose members possessed similar 2D structures, rather than the exact target-ligand binary relationship [6] or similarity of functional ligand sets [7]. A key observation from our study was that although the topology of LCBN and SBN differed, three independent methods partitioned proteins in both networks into nearly the same communities [for detailed list, see Additional file 3]. Actually, the threshold of the similarity for network drawing only affected the found communities a little. We clustered and compared the threshold LCBN and SBN to their corresponding integrate network; we found that the average normalized mutual information was 0.97.

We took targets, pathways, diseases, drug adverse reactions and ligand cluster range of each target into consideration in our website, ePlatton, which could offer the possibility to discern polypharmacology and negative drug reactions. The ePlatton could also be used to predict the target range of the compounds that users interest as used in the validation section. Like some of the most common weaknesses in the ligand-based study and unlike the sequence-based method, the LCBN approach cannot include the protein which does not have relatively high-affinity ligands although its sequence is known.

Throughout the history, pharmaceutical and medical researchers have concentrated on a few target proteins sharing commonalities at the level of sequence and/or ligand. Nowadays, the progress of genomics and chemoinformatics has made it possible to investigate the relationship and communication among large numbers of proteins in the sequence and ligand space. Our specific method could discern the ligand cluster-based organization of targetable proteins in a panoramic view, which could not be easily achieved by studying only a few related ones. It could also provide valuable biological and chemical reference for the lead compound designing.

## Methods

### Protein sequence and ligand 2D structure

All the protein sequences and ligand 2D structures came from ChEMBL_19 data set. We filtered the data by the following conditions: (1) binding affinity value (Ki, Kd, EC50 and IC50) was less than or equal to 10 μm. (2) the molecular weight of ligands was less than 800 Da, to exclude the peptides and other huge molecules. (3) the length of the target protein sequence was more than 80, to avoid the short proteins being highly similar to dozens of other proteins.

### Protein similarity calculation

The sequence-based similarity was obtained by ClustalO (version 1.2.0) [14] with default parameters.

To calculate the ligand cluster-based similarity, we firstly converted the 2D structures of ligands to CDK [12] 2048-bit fingerprints with the depth being 7. The Jaccard similarity [10], which is the same as Tanimoto coefficient (Tc) [29], of every pair of ligands was calculated by Numpy [30]. We used AP cluster (version 2009) [13] to analyse all ligand pairs, whose similarities were above 0.5, with the preference being 0. Then, we got 13,769 ligand clusters. We represented each protein by a bit vector whose length was the same as the number of the ligand clusters (13,769) and if a protein interacted with ligand(s) in *i*th ligand cluster, the *i*th bit in the vector would be 1. In this way, we annotated the proteins by the ligand cluster(s) they interacted and the ligand cluster-based similarity of proteins was indicated by the Jaccard index.

### Networks

The node represented a protein and the edge represented the similarity of two proteins it linked. For the legibility of LCBN and SBN, we only displayed the edges whose similarities were above 0.25. The simplified networks discussed above were also called threshold networks. The perfused force directed algorithm was used for the layout of LCBN and SBN. The colour code indicated the HGNC gene family. The visualization of networks was achieved by Cytoscape [31] and gene family names were labelled by Inkscape.

### Compare both networks

We used AP cluster [13], infoMap from igraph-python (version 0.7.0) [32] and MCL (version 12-068) [33] to find clusters in both integrate and threshold networks. We used the preference at 0 for AP cluster, default parameters for infoMap and the inflation at 4.0 for MCL, other unmentioned parameters were the same as default. For different communities from each cluster method, we calculated the normalized mutual information among LCBN, SBN and HGNC gene family to compare the three methods.

### ePlatton

We used Django and SQLite to set up ePlatton. The protein global sequence and ligand 2D structure search were supported by ClustalO and CDK. jQuery and Cytoscape.js helped to achieve the frontend of ePlatton and the display of LCBN and SBN. Molecular pictures were generated by Open Babel [34].

### ePlatton validation and predictive power

We used the open-source platform [35] to benchmark EXEMPLAR (i.e. ePlatton) and data fusion [22] methods (MAX, 3NN, MPS [21]) and 21 targets with more than 30 actives from the directory of useful decoys (DUD) [23] were tested. For each target, 20 % random selected active ligands were used as training set and clustered with AP method as described above. The similarity of training ligands including exemplar ligands against remaining ligands were calculated. MAX, 3NN, MPS and EXEMPLAR similarity scores were calculated and ranked. Scikit-learn [36] and R [37] were used to calculate AUC and P value of post hoc Friedman’s Test [24, 25]. This simulated virtual screen was repeated 50 times per target.

## Additional files

10.1186/s13321-016-0135-5 Ligand cluster size vs degree. The relationship between the number of interacted ligand clusters and the degree of proteins in LCBN.
10.1186/s13321-016-0135-5 The degree distribution of ligand cluster-based network.
10.1186/s13321-016-0135-5 Three method comparison of the threshold and integrate networks. Three methods (AP cluster, MCL and info map) are applied to find network communities within the ligand cluster- and sequence-based protein network (threshold and integrate) and compare with each other and HGNC gene family classification.
10.1186/s13321-016-0135-5 LCBN giant component analysis. The top 10 items of GO enrichment and KEGG pathway analysis and their p-values.
10.1186/s13321-016-0135-5 The 10 most interacted ligand clusters in the LCBN. Each row represents one ligand cluster, the first CHEMBL ID is the exemplar of that cluster.
10.1186/s13321-016-0135-5 The table of 1st example of ePlatton search result.
10.1186/s13321-016-0135-5 The table of 2nd example of ePlatton search result.

## Abbreviations

HGNC: HUGO Gene Nomenclature Committee
LCBN: ligand cluster-based Network
SBN: sequence-based Network
MPS: pair-wise similarity
KNN: K nearest neighbor
DUD: directory of useful decoy
