# Supplementary figures and images for: Ligand cluster-based protein network and ePlatton, a multi-target ligand finder
Source: J Cheminform. 2016 Apr 30;8:23. doi: 10.1186/s13321-016-0135-5 (PMC4853874; doi:10.1186/s13321-016-0135-5)

Ligand cluster size - Degree

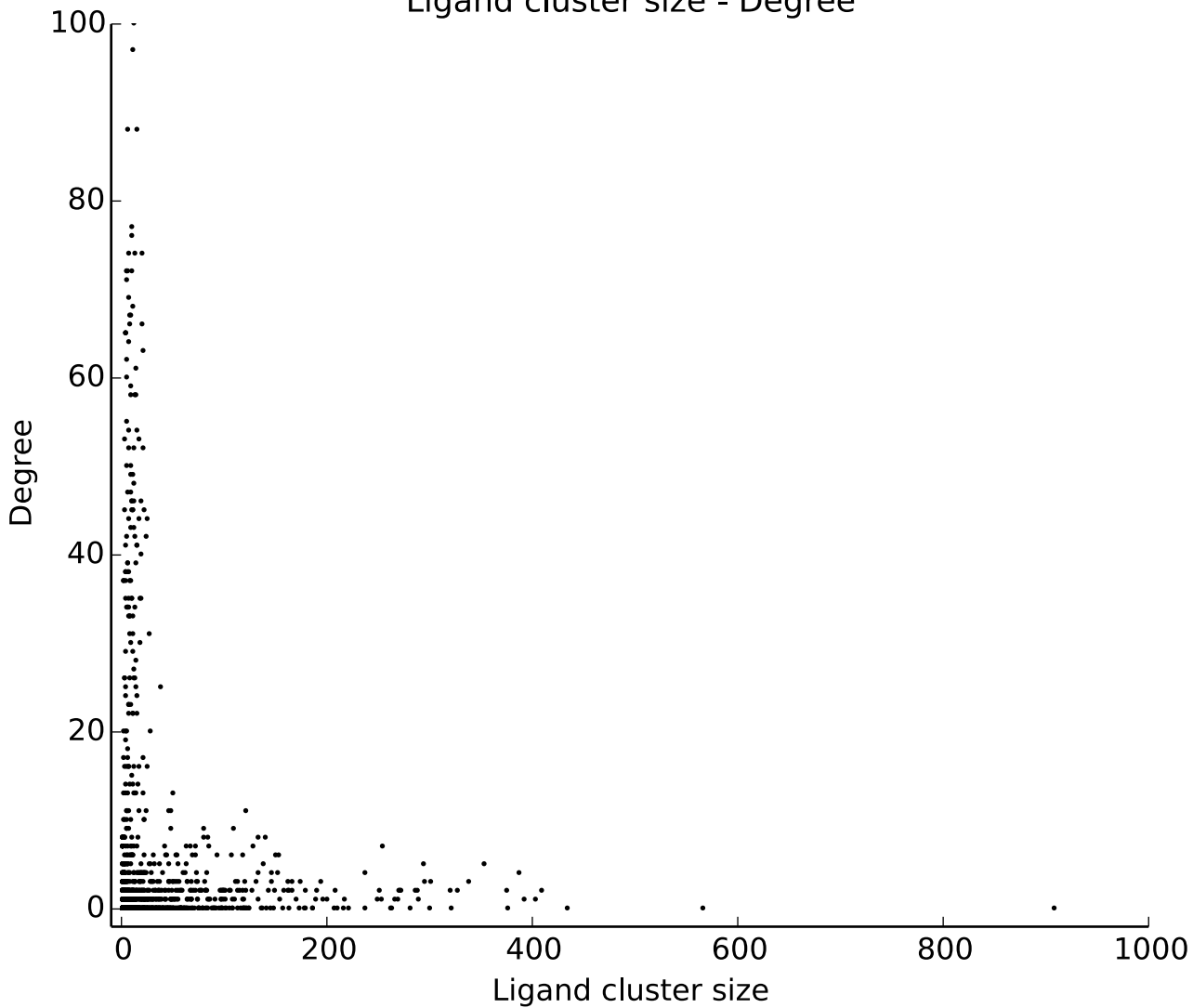

Supplement: Supplementary file 1 — 10.1186/s13321-016-0135-5 Ligand cluster size vs degree. The relationship between the number of interacted ligand clusters and the degree of proteins in LCBN. [file 13321_2016_135_MOESM1_ESM.pdf]

Ligand cluster-based network degree distribution

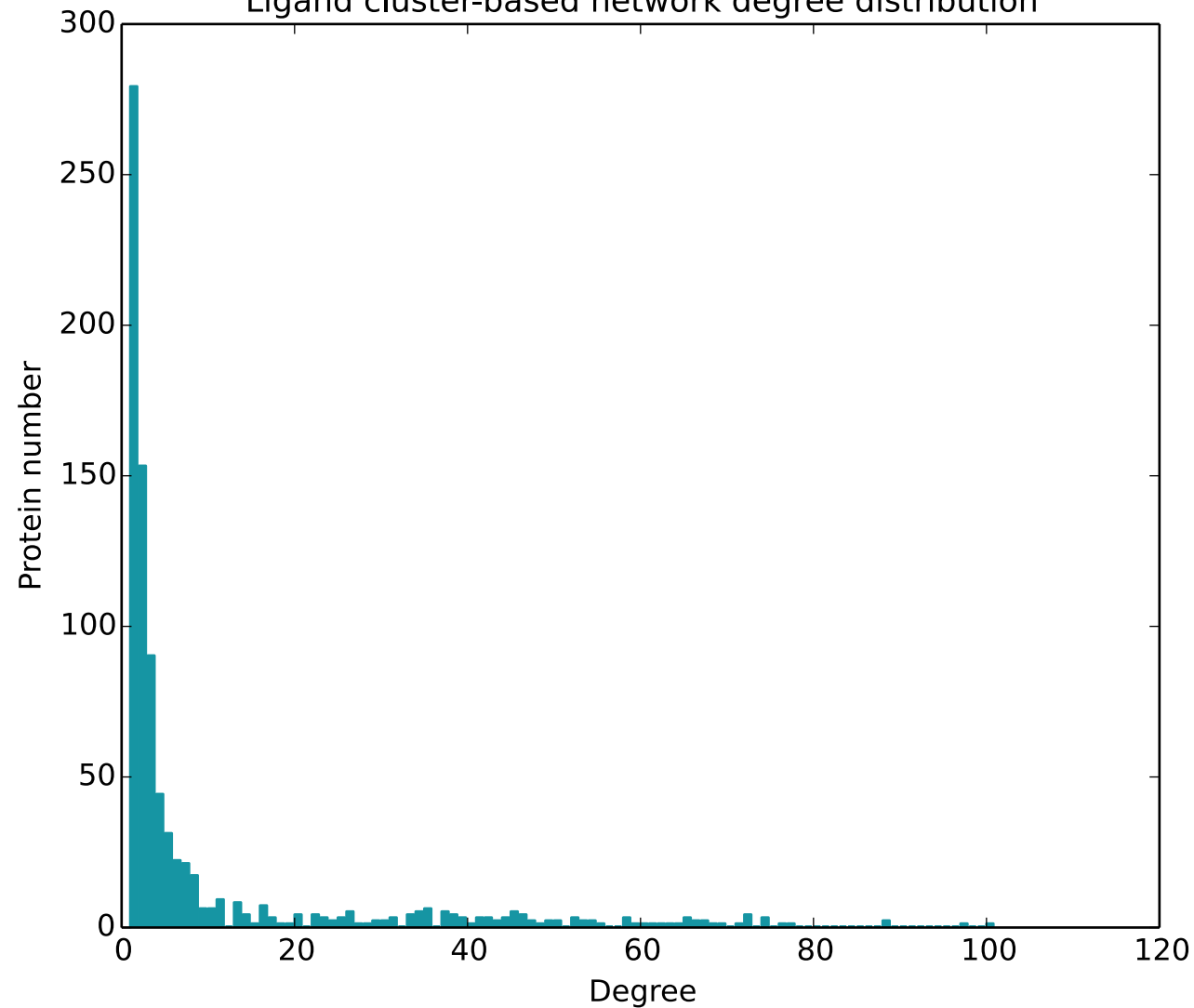

Supplement: Supplementary file 2 — 10.1186/s13321-016-0135-5 The degree distribution of ligand cluster-based network. [file 13321_2016_135_MOESM2_ESM.pdf]
